# The Neuroses of the Genito-Urinary System

**Published:** 1890-07

**Authors:** 


**The Neuroses of the Genito-Urinary System in the Male,** with Sterility and Impotence. By Dr. R. Ultzman, Professor of Genito-Urinary Diseases in the University at Vienna. Translated by Gardner W. Allen, M. D., Surgeon in the Genito-Urinary Department, Boston Dispensary. Philadelphia and London: F. A. Davis. 1889. Price, $1 net.

This interesting subject is handled by this author in a clear and perspicuous manner. Since attention has been, of late, directed to the importance of these diseases and the relation they have to sterility on both sexes, every bit of literature bearing thereupon is in demand.

Dr. Ultzman is so well known as an author and a teacher that whatever he puts forth is sure to attract attention, and he has done himself credit and the profession a service in the present instance. There are yet many unsettled questions bearing on the subjects treated of in this book, and the author has contributed something towards their solution. He has not assumed to speak dogmatically where there is still doubt, but has given the results of his own observations upon most points. It is a book that cannot fail to find ready sale.

